# Challenging retrieval of a dislodged leadless pacemaker from the left pulmonary artery in an older adult patient

**DOI:** 10.1016/j.hrcr.2025.02.015

**Published:** 2025-02-18

**Authors:** Yuya Nakamura, Kengo Suzuki, Yumi Yamamoto, Yoshimi Onishi, Taku Asano, Toshiro Shinke

**Affiliations:** Division of Cardiology, Department of Medicine, Showa University School of Medicine, Tokyo, Japan

**Keywords:** Pacemaker, Device dislodgement, Device retrieval, Older adult patient, Atrioventricular block, Pacemaker complication

**Key Teaching Points**

• Early recognition of leadless pacemaker instability is crucial to prevent complications.
• Signs such as "dancing Micra" behavior and low impedance values should prompt timely intervention to avoid migration and associated risks.
• Advanced tools enable safe retrieval of dislodged leadless pacemakers in complex anatomy.
• The combined use of the Agilis NxT sheath (Abbott, Tokyo, Japan) and EN Snare (Merit Medical Japan, Tokyo, Japan) facilitated precise navigation and successful retrieval from the left pulmonary artery.
• Efficient procedural techniques minimize patient risks and radiation exposure.
• Optimized techniques reduced the fluoroscopy time to 282 seconds and the radiation dose to 7 Gy · cm^2^, ensuring procedural safety for high-risk patients.
• Leadless pacemaker retrieval is feasible and safe even in older adult patients.
• This case demonstrates the effectiveness of advanced retrieval techniques in older adult patients, with a successful 2-year follow-up confirming long-term stability.

## Introduction

Micra AV leadless pacemakers (Micra, Medtronic, Tokyo Japan) offer significant benefits, even in older adult patients.^1^ However, device dislodgement into difficult-to-reach areas, such as the left pulmonary artery (LPA) requires advanced techniques and appropriate tools for retrieval.^2^

## Case report

A 92-year-old man with progressive dyspnea on exertion was diagnosed with heart failure secondary to a 2:1 atrioventricular block. His medical history revealed hypertension and dyslipidemia. Transthoracic echocardiography revealed preserved left ventricular function with an ejection fraction of 62% and no significant valvular abnormalities. Due to the patient’s frailty, advanced age, and comorbidities, a Micra AV leadless pacemaker was selected for permanent pacing to avoid pocket- and lead-related complications.

### Device implantation and migration

**Figure 1:**
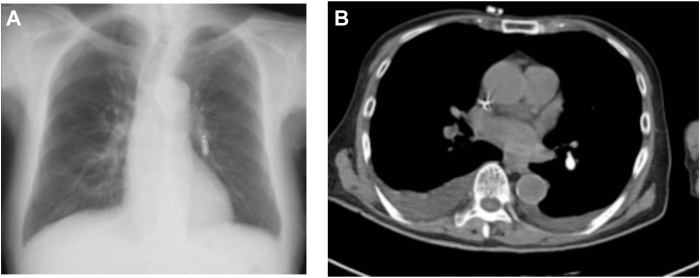
**A:** Chest radiograph showing the dislodged Micra pacemaker (Medtronic, Tokyo, Japan) in the left pulmonary artery (LPA). **B:** Computed tomography image showing the Micra pacemaker embolized into a branch of the LPA.

The Micra was successfully implanted at the right ventricular septum after 2 deployment attempts. The device was confirmed to be securely fixed with 2 tines. The pacing threshold was 0.75 V (0.24 ms), with an R-wave amplitude of 7.2 mV and impedance of 480 Ω. However, pacing failure was detected on postoperative day 1. Imaging, including chest x-ray and computed tomography (CT), confirmed migration of the Micra to the lower branches of the LPA (Figure 1A and 1B).

### Pre-retrieval assessment

**Figure 2:**
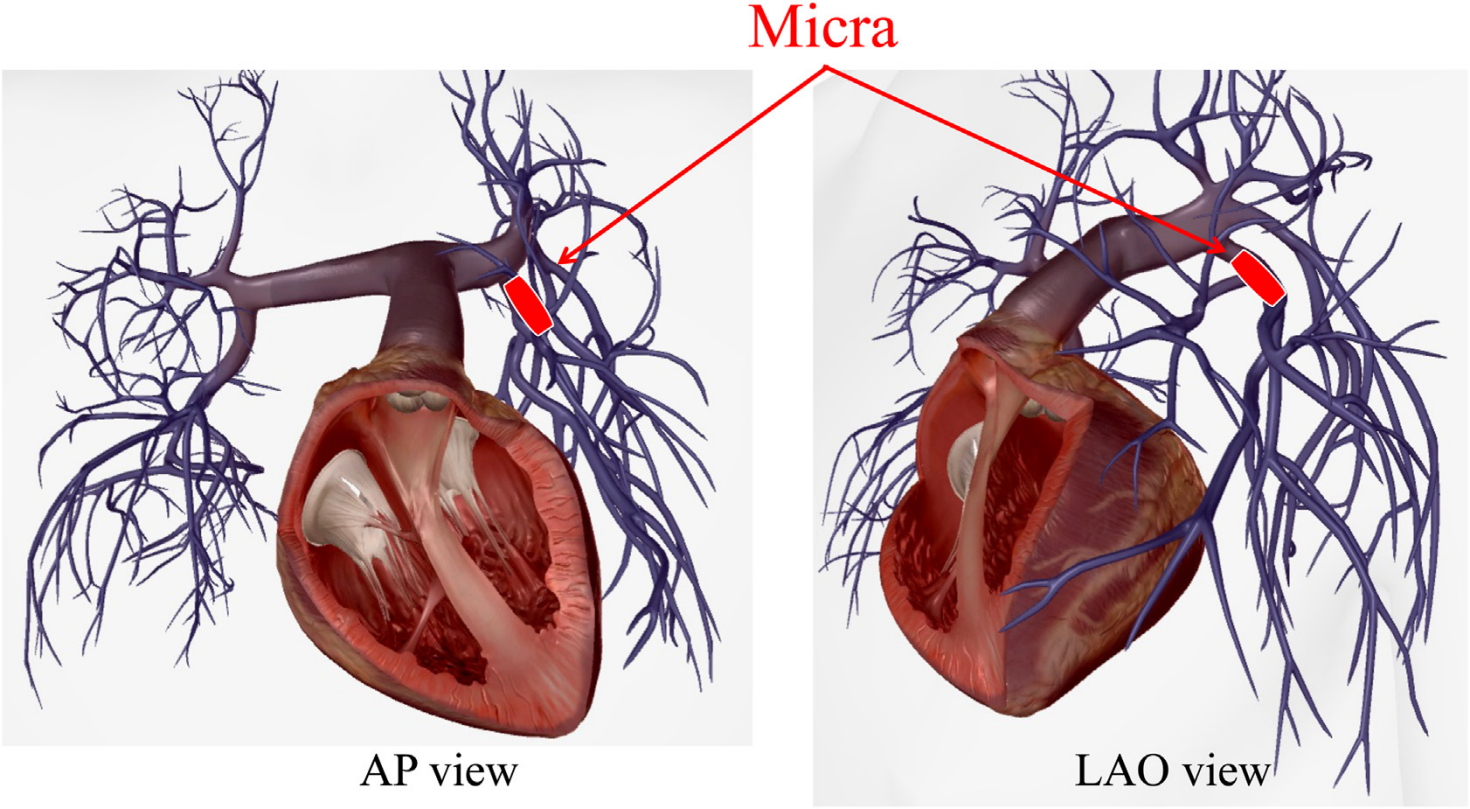
Schematic representation of the pulmonary artery showing the embolization of the Micra pacemaker (Medtronic, Tokyo, Japan) in the lower lobe branch of the left pulmonary artery. The image was created using the Human Anatomy Atlas (version 2025.0.12, Visible Body). AP = anteroposterior. LAO = left anterior oblique.

**Figure 3:**
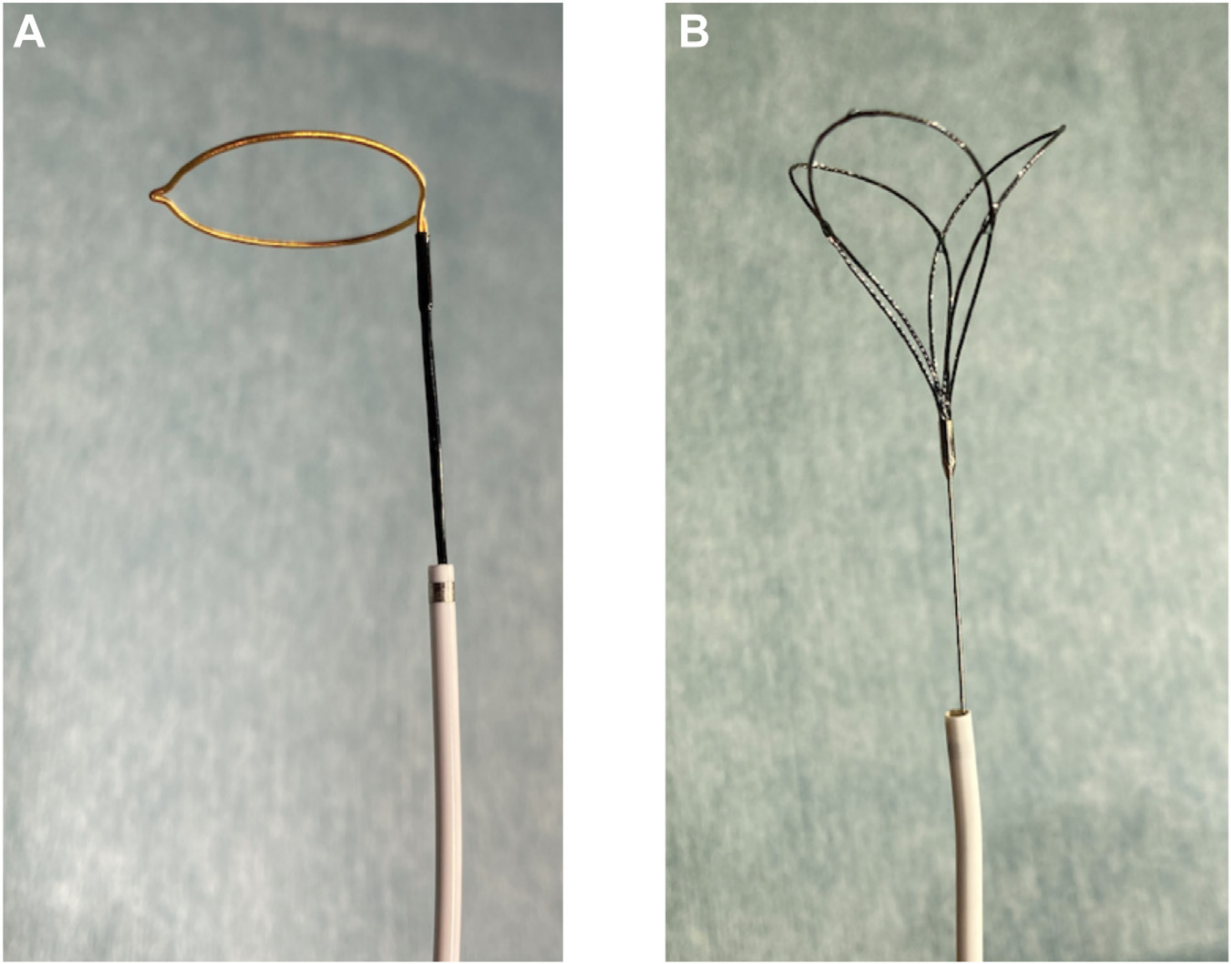
**A:** Photograph of the ONE Snare (Merit Medical, Tokyo, Japan), a single-loop snare device that was initially used for Micra leadless pacemaker retrieval in this case. **B:** Photograph of the EN Snare, a triple-loop snare device designed to expand 3-dimensionally, prepared for use due to the anticipated challenges of capturing the device in the narrow branches of the left pulmonary artery.

Pre-retrieval imaging using CT confirmed that the Micra had embolized to the LPA, with the retrieval head located distally and the tines positioned proximally. The LPA was expected to pose difficulty in catheter navigation, as the catheter inserted from the inferior vena cava would likely bend twice, creating an S-curve, which could complicate access to the LPA (Figure 2). Therefore, Agilis NxT sheath (Abbott, Tokyo, Japan) was planned for use in the retrieval procedure. The narrow lumen of the LPA branches was expected to hinder reliable device capture with conventional snares, so a triple-loop snare, the EN Snare (Merit Medical Japan, Tokyo, Japan), was also prepared (Figure 3A and 3B). The retrieval of the Micra carried risks of vascular and cardiac injury. Therefore, a heart team conference, including cardiovascular surgery and anesthesiology, was held to discuss the treatment plan, and backup support was arranged in case of complications during the procedure. The decision was made to proceed under general anesthesia to stabilize respiration and ensure a prompt response in the event of any complications.

### Retrieval procedure

#### Access and setup

The Micra introducer was inserted through the right femoral vein. The retrieval procedure was attempted by inserting a snare through a Judkins right (JR) catheter, as suggested in previous reports.^3^ However, the JR catheter, due to its natural curvature, primarily advanced toward the right pulmonary artery, and even when it reached the LPA, it was unable to select the downward branching. To overcome that anatomic challenge, the Agilis sheath was inserted through the left femoral vein for retrieval. To minimize cardiac injury while navigating the right ventricular outflow tract, a JR catheter was placed inside the Agilis sheath and advanced to the pulmonary artery as an anchor. Under fluoroscopic guidance, the Agilis sheath was carefully advanced in small steps through the S-curve, with the JR catheter ensuring stability. The sheath tip was controlled to avoid the larger curve of the bend and positioned safely at the pulmonary artery trunk.

#### Retrieval process

**Figure 4:**
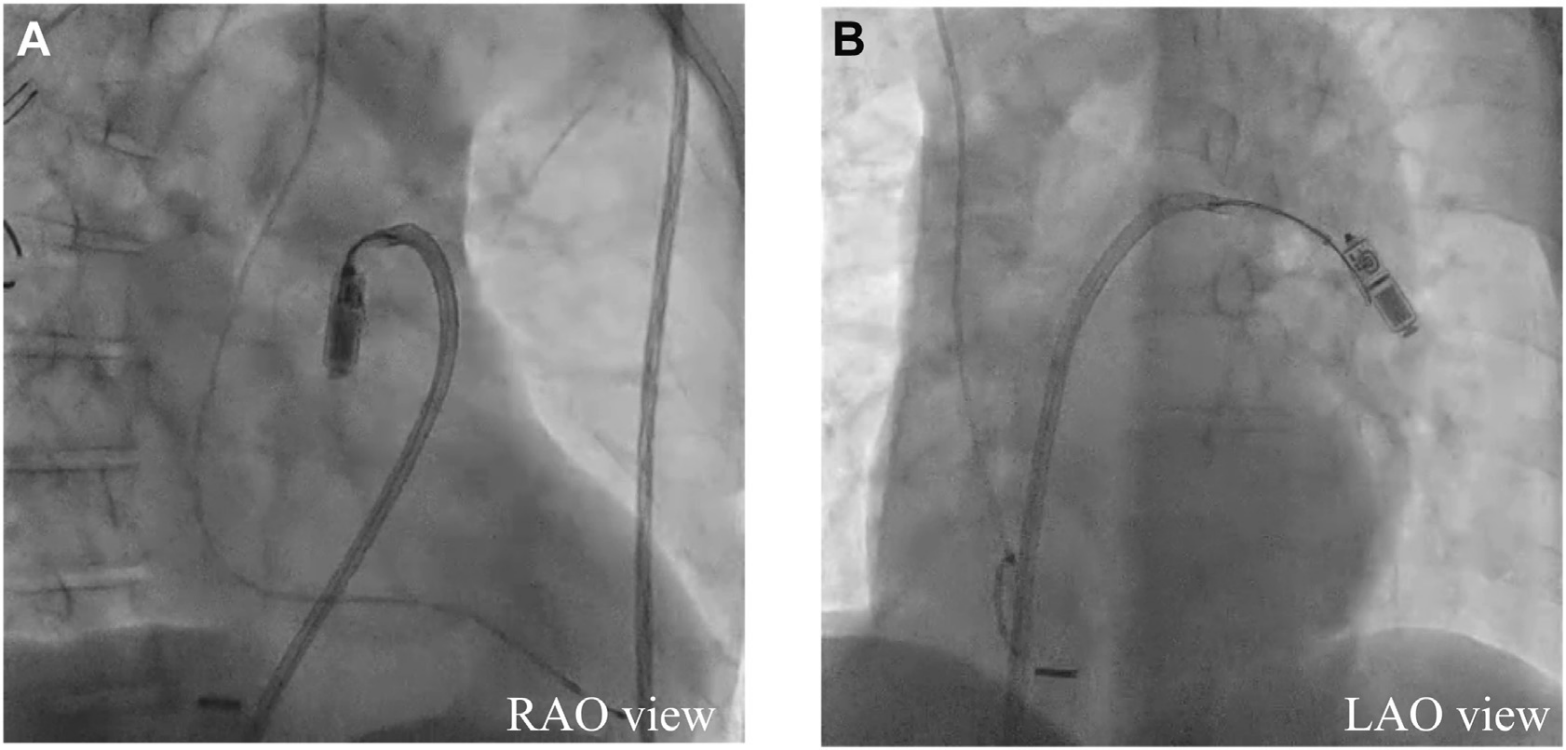
Fluoroscopic images in right anterior oblique (RAO) (**A**) and left anterior oblique (LAO) (**B**) projections. The images demonstrate the Agilis sheath (Abbott, Tokyo, Japan) advanced to the pulmonary artery trunk and deflected to access a lower branch of the left pulmonary artery. The EN Snare (Merit Medical, Tokyo, Japan) is capturing the tines of the embolized Micra device (Medtronic, Tokyo, Japan) for retrieval.

**Figure 5:**
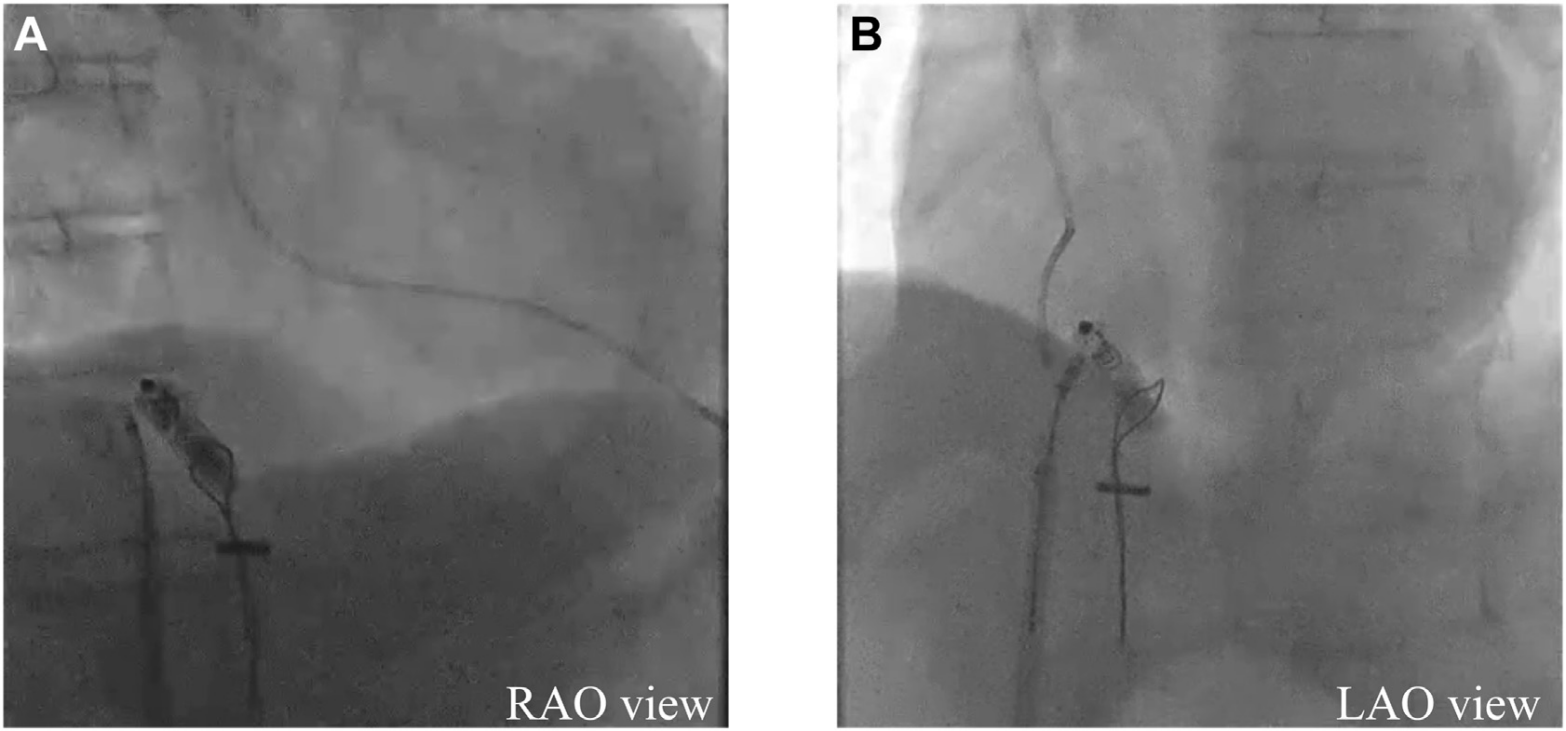
Fluoroscopic images in right anterior oblique (RAO) (**A**) and left anterior oblique (LAO) (**B**) views demonstrating the sequential steps of device retrieval. The EN Snare (Merit Medical, Tokyo, Japan) is used to grasp the tines of the dislodged Micra device (Medtronic, Tokyo, Japan) and pull it into the right atrium. Subsequently, the ONE Snare (Merit Medical, Tokyo, Japan), introduced via the Micra introducer, secures the retrieval head for controlled extraction.

Given the narrow caliber of the LPA branches, conventional snares were insufficient for reliable device capture. The EN Snare was deployed, achieving 3-dimensional opening within the vessel and providing a broad and stable capture area. Direct capture of the proximal tine proved challenging. The EN Snare loop was partially tightened around the midsection of the Micra and gently pulled, successfully securing the proximal tine (Figure 4A and 4B). The EN Snare was used to grasp the tines and pull the device into the right atrium (Supplemental Video 1). To complete the retrieval, a double-snare technique was used in the right atrium.^4^ Using a second snare introduced through the Micra introducer, the retrieval head was firmly grasped, enabling precise control and smooth extraction of the Micra into the sheath (Figure 5A and 5B).

#### Post-retrieval and reimplantation

After retrieval, a new Micra was implanted at the right ventricular septum. Fixation with 3 tines was confirmed, and the absence of "dancing Micra" was verified. The pacing threshold was 0.5 V (0.24 ms), R-wave amplitude 8.8 mV, and impedance 780 Ω. The total procedure time was 52 minutes, including 282 seconds of fluoroscopy, with a radiation dose of 7 Gy · cm^2^. Post-retrieval CT confirmed complete removal of the dislodged device without residual fragments or evidence of vascular injury. Transthoracic echocardiography confirmed no significant changes. Stable electrical parameters were achieved, and the procedure was completed without complications.

#### Follow-up and outcomes

The patient’s postoperative course was uneventful. Heart failure management and rehabilitation were carefully conducted due to advanced age, enabling the patient to walk independently. He was discharged on postoperative day 19 with heart failure classified as New York Heart Association class I. During a 2-year follow-up with evaluations every 3 months, echocardiography showed no abnormalities. The pacing threshold remained stable at 0.5–0.75 V (0.24 ms), and the device demonstrated consistent performance without migration, threshold elevation, or battery depletion.

## Discussion

### Considerations for retrieving migrated leadless pacemakers from the pulmonary artery

Li and colleagues^5^ recommended retrieving migrated leadless pacemakers to prevent complications like thromboembolism. Based on this report, we chose retrieval to avoid long-term risks. Although forgoing retrieval may be considered in asymptomatic cases, leaving the device in the pulmonary artery poses risks of vascular injury or infection. Retrieval of leadless pacemakers from the pulmonary artery branches is inherently difficult due to their narrow caliber and sharp bifurcations. In our case, the LPA presented additional complexity, as the catheter had to navigate a significant S-curve when inserted from the inferior vena cava. Potential complications, including pulmonary artery perforation and thrombosis, were carefully evaluated. In this case, the tines were positioned proximally and the retrieval head distally within the pulmonary artery, reducing the risk of vascular injury during retrieval. However, had the positions been reversed—that is, the retrieval head proximally and the tines distally—the sharp edges of the tines could have posed a significant risk of vascular injury and made retrieval more challenging. In such situations, if the patient remains asymptomatic, forgoing retrieval could be a reasonable option to avoid unnecessary procedural risks.

### Innovative techniques in this case

To overcome these challenges, we used 3 key innovations.

#### Safe advancement and selectivity of the Agilis sheath

**Figure 6:**
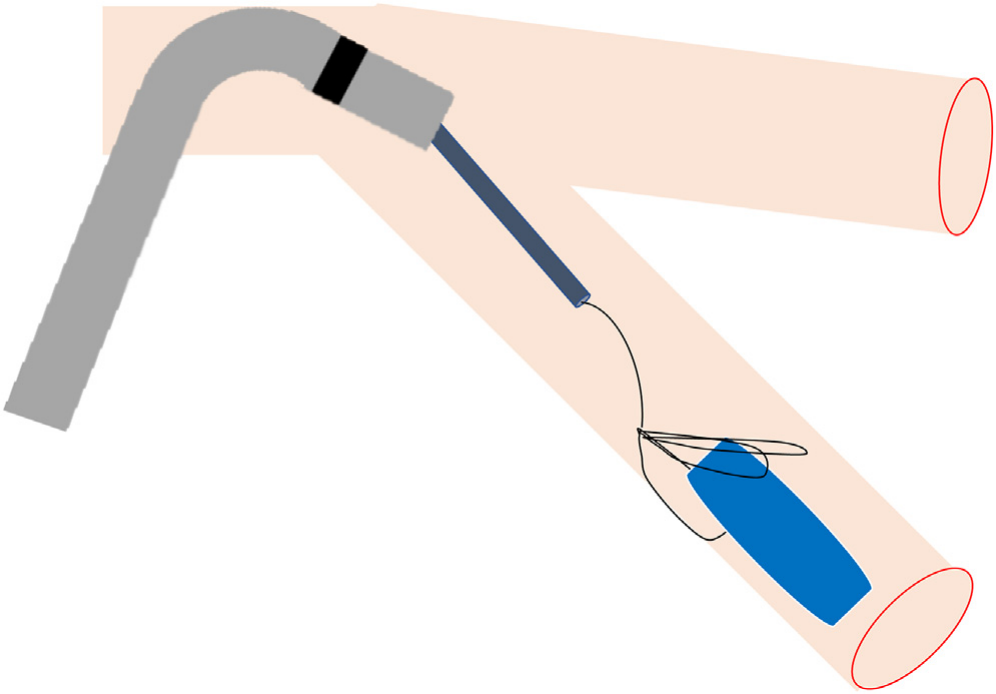
Schematic illustration demonstrating the use of the Agilis sheath (Abbott, Tokyo, Japan) to select a lower branch of the left pulmonary artery. The EN Snare's (Merit Medical, Tokyo, Japan) 3-dimensional loop expansion facilitates secure grasping of the dislodged Micra device (Medtronic, Tokyo, Japan), enabling efficient retrieval.

By gradually adjusting the deflection of the Agilis sheath, we were able to safely navigate through the S-curve and reach the main pulmonary artery without causing trauma to the vessel wall. The deflection of the Agilis sheath allowed for successful selection of the inferior branch of the LPA and was also useful in maintaining coaxial alignment between the EN Snare and the Micra device (Figure 6).

#### EN Snare for 3-dimensional capture

Given the narrow caliber of the LPA branches, conventional snares were insufficient for reliable device capture. The EN Snare was deployed, achieving 3-dimensional opening within the vessel and providing a broad and stable capture area. Direct capture of the proximal tine proved challenging. The EN Snare loop was partially tightened around the midsection of the Micra and gently pulled, successfully securing the proximal tine.

#### Grasping the tines for retraction and the retrieval head for insertion

During the pre-retrieval assessment, we planned to grasp the proximal tines to retract the device to the right atrium and then re-grasp the retrieval head to insert it securely into the Micra introducer. This approach was based on the consideration that grasping the tines for retraction would prevent pulmonary artery and cardiac injury, while attempting to insert the device into the Micra introducer using the tines alone might increase the risk of dropping the device. For this reason, the Agilis sheath was inserted through an independent access point, separate from the Micra introducer, and the retrieval was performed by regrasping the device using the double snare technique. This method proved to be highly effective for safe and timely retrieval.

### Lessons learned from the initial Micra implantation and its migration

The initial Micra implantation provided valuable insights into potential early indicators of suboptimal fixation. Although the pacing threshold was initially acceptable at 0.75 V (0.24 ms), the impedance value of 480 Ω was insufficient, indicating inadequate fixation.^6^^,^^7^ Retrospectively, this low impedance could have been an early warning sign of device instability, ultimately leading to migration. In addition, after the tether was cut, the Micra displayed slight "dancing Micra" behavior, characterized by unstable movements due to insufficient anchoring (Supplemental Video 2). This instability warranted early retrieval and reimplantation to prevent complications. Addressing these signs promptly might have prevented the device's migration to the LPA and ensured a more stable outcome. Furthermore, recognizing these early warning signs helped refine our approach for the second implantation. By addressing anatomic challenges and considering Micra’s benefits for older adult patients, we chose the Micra device again. This case highlights the importance of close monitoring during leadless pacemaker implantation. Careful assessment of pacing thresholds, impedance values, and any signs of device instability, such as "dancing Micra," is critical for ensuring optimal fixation and long-term device stability.

### Safety, feasibility, and clinical implications

This case demonstrates the safety and feasibility of using the Agilis sheath and EN Snare for retrieving dislodged Micra devices in high-risk older adult patients. Although previous reports have described the use of JR catheters for retrieving leadless pacemakers, in this case, navigating to the lower lobe branch of the LPA using the JR catheter was not feasible due to the complex anatomic configuration. To address this limitation, the Agilis sheath was advanced to the main pulmonary artery, providing stable support and enabling precise navigation. Furthermore, the EN Snare, with its 3 fully deployed loops, created a 3-dimensional capture mechanism that ensured efficient and safe retrieval of the dislodged pacemaker. This innovative approach highlights the potential applicability of these techniques in anatomically challenging cases, offering a valuable alternative to conventional methods. The procedure was completed without complications, with minimal radiation exposure (282 seconds of fluoroscopy, 7 Gy · cm^2^) and a total time of 52 minutes. Imaging confirmed complete device removal without vascular injury, and a new Micra was successfully reimplanted, maintaining stable pacing parameters over a 2-year follow-up. The technique proved effective in navigating complex anatomy and achieving secure device retrieval with minimal complications. Advancing the Agilis sheath to the pulmonary artery trunk, however, may carry risks and should be reserved for selected cases. Notably, in this case, the snare advanced through a JR catheter was able to easily reach branches in the upper lobes of both the right and left pulmonary arteries. For Micra embolizations in such branches, retrieval using a snare alone, without relying on the Agilis sheath, may be a safer alternative. The described technique demonstrates both adaptability and reliability, making it a valuable option for managing challenging clinical scenarios.

## Conclusion

The Agilis sheath and EN Snare provide an effective and safe method for retrieving dislodged Micra pacemakers, even in challenging anatomic locations and older adult patients. This technique enables rapid retrieval with minimal complications and demonstrates potential for broader clinical applications in similar challenging scenarios.

## Disclosures

The authors have no conflicts of interest to disclose.
