# Supplementary material for: Challenging retrieval of a dislodged leadless pacemaker from the left pulmonary artery in an older adult patient
Source: HeartRhythm Case Rep. 2025 Feb 18;11(5):467–72. doi: 10.1016/j.hrcr.2025.02.015 (PMC12242977; doi:10.1016/j.hrcr.2025.02.015)
Supplement: Supplementary Data [file mmc3.docx]

**Supplementary Legends**

Supplementary Video 1: The EN Snare was used to grasp the tines and pull the device into the right atrium.

Supplementary Video 2: After the tether was cut, the Micra displayed slight "Dancing Micra" behavior, characterized by unstable movements due to insufficient fixation.
